# Extraction and separation feasibility of cerium (III) and lanthanum (III) from aqueous solution using modified graphite adsorbent

**DOI:** 10.1007/s11356-022-20823-9

**Published:** 2022-06-17

**Authors:** Maha A. Youssef, Nesreen M. Sami, Hisham S. Hassan

**Affiliations:** Hot Laboratories Center, Egyptian Atomic Energy Authority, P.O. Box 13759, Cairo, Egypt

**Keywords:** Alginate, Graphite, Lanthanum, Cerium, Adsorption

## Abstract

Graphite (GR) and graphite/alginate (GRA) composite were synthesized utilizing the thermal annealing technique and used as a new adsorbent material for the selective separation and removal of La(III) and Ce(III) from aqueous solutions. Fourier transform infrared (FTIR) spectroscopy, thermal analysis (DTA, TGA), X-ray diffraction (XRD), surface area, porosity, and scanning electron microscope (SEM) were also used to characterize the generated material. Distinct experiments were performed to test the ability of the GRA to La(III) and Ce(III) removal, which include the effect of pH, shaken time, initial concentration of La(III), and Ce(III) at different temperatures range. After 20 min, both ions have reached equilibrium. The pseudo second-order kinetic model was chosen as one which best fits the experimental evidence and better reflects the chemical sorption process. Adsorption isotherm was studied using the Langmuir, Freundlich, and D-R models. The Langmuir model was used to better fit the results obtained. At 25 °C, Ce(III) and La(III) have maximum monolayer capacities of 200 and 83.3 mg/g, respectively. The sorption was endothermic reaction and spontaneous, as illustrated by the data of thermodynamics studies. GRA has the ability to be used as a novel lanthanide adsorbent material, especially for selective separation between Ce(III) and La(III).

## Introduction

A contaminant material displays a substantial challenge for many scientists to protect humanity and the environment from the risky effect (Allam et al. 2021). The contaminants have multiple sources in the environment as organic dyes, radioisotopes, and heavy elements (Wu et al. 2019; Hassan et al. 2019). The radioisotopes are produced from many applications such as nuclear fuel, medical diagnoses, and treatment (Zhang et al. 2019; Abdel Maksoud et al. 2022). One of the fission products generated by irradiated nuclear fuel is lanthanides elements (Vergas et al. 2021; Lankapati et al. 2021). Also, lanthanide elements have a distinct application in many fields, for example, metallurgy and the ceramic industry. Therefore, the demand for lanthanides will be an utmost in the future, particularly using the high purity lanthanides in high technology applications (Zhu et al. 2015; Wang et al. 2013). The waste containing lanthanide elements is discharged into the environment, so their removal from respective mixtures has considerable importance and their recoveries are very important (Lee et al. 2005; Bonal et al. 1998; Volokh et al. 1992).

Many techniques were used for the separation and extraction of lanthanide elements such as adsorption, liquid–liquid extraction, membrane, ion exchange, and solid–liquid extraction (Gupta and Krishnamurthy 1992; Li and Sun 2005; Hassan and Elmaghraby 2019). One of the most efficient separating techniques is the adsorption process (Ahmadi et al. 2022; Naeini et al. 2022; Moradi and Sharma 2021; Babudurai et al. 2021). This method has a number of advantages, including fewer wastes and a faster solid–liquid separation. Several bio adsorbents have received a lot of attention for adsorption in the previous decade, owing to their low cost and environmental integrity (Moradi et al. 2021; Kim et al. 2011; Ngah and Hanafiah 2008; Zhang et al. 2012; Xiong et al. 2011; Gupta et al. 2015). Alginate acts as a biologically degradable carbohydrate polymer that is often produced from brown seaweed, and it is the sodium salt of alginic acid (Kazemi and Javambakht 2019). It is composed of α–L–G (guluronic acid) with β–D–M (mannuronic acid). The effective functional groups in alginate are carboxylic and hydroxyl groups (Huixue et al. 2016; Zj et al. 2018). Alginate has powerful binding abilities and hydrophilicity properties as well as biologically renewable for many hazardous elements removal (Cajnko et al. 2019; You et al. 2019). Encapsulation of particles with alginate beads via calcium ion cross-linking is a less hazardous impregnating approach (Anisha and Prema 2008; El-Din et al. 2021). Alginate adsorbents have restricted use because of their low mechanical strength and durability. Combining alginates with other materials such as graphene oxide (Mirzaeian et al. 2019; Malinga and Jarvis 2020), activated carbon or graphite with suitable particle size distribution and large pores, high adsorption capacity, and specific surface area (Ahmadi et al. 2021) has improved their mechanical qualities (Rajabiet al. 2017). Rajendran et al. (2021) reported that carbon-based materials and their composites are super capacitors due to their high efficiency, superior energy densities, low cost, and prolonged life span (Rajendran et al. 2021).

Graphite with its considerable surface area and microporous nature is promising adsorbent for the removal of hazardous elements from wastewater. The applications of porous graphite for the removal of hazardous elements can be restricted by slow diffusion kinetics) Pattanayak et al. 2000; Pelekani and Snoeyink, 1999), but graphite nanostructure has found its place as an alternative of the porous graphite materials. The properties of nano graphite make them promise nuclear technology for the removal of hazardous elements (Iuima 1991; Hussain and Iqbal 2011). Babaei et al. (2021) prepared a quantum dots adsorbent using carbon-based materials (grapheme) with particle size less than 10 nm as a super adsorbent for heavy metals removal from waste water (Babaei et al. 2021).

Olatunji et al. (2018) used a sorption approach and a composite of polypyrrole and wood sawdust to remove ^139^Ce (a strong gamma-emitting radioisotope of cerium with *T*_1/2_ = 137.641 day). According to the Langmuir model, the maximal capacity was found to be 6.57 ± 0.54 mg/g. Sert et al. (2008) investigated the biosorption of La(III) and Ce(III) by Platanus oriental leaf. According to the results, the sorption was endothermic in nature and spontaneous, with the adsorbent’s Langmuir capacity of 28.65 mg/g for La(III) and 32.05 mg/g for Ce(III) (Sert et al. 2008).

The main objective of this study is to prepare high performance and high sorption capacity adsorbent material (graphite/alginate) for selective separation and removal of some lanthanides (Ce III and La III) from aqueous solution. The sorption was discussed in terms of pH, contact time, concentration, temperature, and interfering ion. Kinetic, thermodynamic parameters, and isotherm studies related to the process were performed.

## Experimental

### Materials and methods

All used reagents in the present study are analytical grade and used without any additional purification. The used cerium chloride and lanthanum chloride were purchased from Fluka Germany Company. Sodium alginate, C_5_H_7_O_4_COONa, with a molecular weight of 216.12, and calcium chloride were purchased from Sigma-Aldrich. The pH was adjusted by the addition of NaOH/HCl acquired from Fluka.

### Instrumentation

The initial and final concentrations of Ce (III) and La (III) were determined by a Shimadzu UV–visible spectrophotometer model UV-160 A, Japan. The pH of the solution was measured by a pH meter (Thermo Scientific). The morphological features of graphite/alginate (GRA) adsorbent before and after adsorption of Ce^III^ and La^III^ were recorded by scanning electron microscope (Philips XL 30). FT-IR characterization was carried out by spectrometer model 2000 FTIR, Perkin Elmer Company, USA, covered the range from 400 to 4000 cm^−1^. The crystal structures of pure graphite, alginate, and graphite/alginate (GRA) were analyzed by Shimadzu X-ray diffractometer (XRD) model XD-Dl, Kyoto, Japan. The measurements of the phase changes and weight losses of GRA were performed with the Shimadzu DTA–TGA system of type DTA-TGA-60 H, Japan.

### Preparation of graphite (GR)

Synthetic routes of GR in detail, pure acrylamide (AA) powder, and ammonium persulfate (APS) were used in the preparation of the organo-sulfonicacids. Each 56.9 g (0.8 mol) of AA monomer was dissolved in double-distilled water up to 200 mL. A hot plate stirrer was used to dissolve the monomer in water at room temperature at a low frequency. The stirrer was adjusted to heat the solution within the reaction flask to a temperature of 80 °C. The initiator was dissolved in water so that 0.1 mol/L of APS was heated to 80 °C. Mixing of the APS solution and the AAmonomer solution took place at 80 °C. The blend was stirred heating for 10 min to ensure complete mixing. In the present work, 1 mL (0.001 mol) of APS solution was added to 20 mL (0.08 mol) of AA solution, i.e., to have a molecular monomer to initiator ratio of 80:1. Only 20 mL of the monomer was used in the preparation of the gel to avoid the overheating that occurs during the exothermic polymerization reaction. Using large amounts of monomer solution would require special attention and the use of a heat sink setup. Polymerization occurs with complete consumption of the monomer to form the polymers, i.e., there are no effective termination reactions. After the preparation of the gel, the reaction process continued for a long time to degrade the properties of the prepared samples. After preparation of the gel, samples were left to dry at 60 °C for 5 days to ensure the full consumption of the monomer and solidification of the gel. Samples were treated thermally at 250 °C for 3 h at ambient temperature. After annealing, samples were left overnight to complete the thermolytic reaction at room temperature. Then the samples were smashed and annealed at 350 °C for an extra 3 h and with extra annealing at 460 °C for 3 h to stimulate the formation of graphite structures.

### Preparation of Ca-alginate/graphite (GRA) adsorbent

The graphite nanostructures were made by thermolysis of a synthesized poly-acryl amide gel (Hassan and Elmaghraby 2012). The alginate 2% (w/v) was synthesized by dissolving Na-alginate)2 g) in distilled water (100 ml) with constant stirring strength (300 rpm) until a gel solution was obtained. (GRA) beads were syntheses by blending 1 g graphite with (100 ml) of Na-alginate gel 2% (w/v); the solution was kept under agitation until a homogeneous gel was obtained which was then slowly added drop wise through a peristaltic pump at 3.5–4.0 rpm into 200 ml of 2% (or 0.2 mol/L) aqueous calcium chloride solution at 25 °C with gentle stirring. Then, the sphere became compact after 2 h of drying, and the size of the resultant beads was measured by a sieve (1.5 + 0.2 mm). The gel beads were rinsed with distilled water several times to remove free Ca(II) and dried in the air at 25 °C for 24 h.

### Adsorption experiment

Adsorption experiments of La(III) and Ce(III) on GRA and GR were carried out in 50-mL conical tubes at a 100 rpm mixing rate and 25 °C in a water bath shaker. A total of 0.01 g of adsorbent was blended with 10 mL solutions containing known concentrations of La(III) and Ce(III) in the tubes to perform adsorption kinetic studies. Samples were collected and filtered with a 0.45-mm syringe filter (Pall Co., USA) at predetermined time intervals to evaluate the dissolved ionic concentrations of the residual metal ions in the solutions. Adsorption isotherm experiments were performed at different temperatures with an initial concentration of 50–250 mg/L. The removal % (R%) (Mozaffari et al. 2020a, b, c) and the adsorption capacity (Sheshdeh et al. 2014) (q_e_ (mg/g)) were determined according to the following Eqs. (1) and (2), respectively:

1 $$R\%=\frac{{\complement }_{0}-{\complement }_{e}}{{\complement }_{e}} \times 100$$

2 $${q}_{e }= \frac{\left({C}_{0}-{C}_{e}\right)V}{m}$$

The starting and equilibrium concentrations of adsorbate (mg/L) are C0 and Ce, respectively. V (L) and m (g) denote the volume of the aqueous solution and the mass of the adsorbent, respectively. The concentration of lanthanide metal ion was determined spectrophotometrically by Arsenazo (III) method. The samples were analyzed at wavelength 650 using a Shimadzu model 160A double-beam UV spectrophotometer.

### Determination of lanthanides elements

Initial and equilibrium lanthanide concentrations in the aqueous phase were estimated by a spectrophotometric method using arsenazo (III) as a reagent. It is used for spectrophotometric determination of rare earth elements (REEs), in acid solutions, arsenazo (III) has a purplish-red color while at higher pH values, it is blue-violet. The absorbance of free arsenazo III (*λ* max = 520–530 nm) at the absorption maxima of the metal complexes (*λ* = 655–675 nm) is very slight. This large difference (Δλ) between the wavelength of the absorption maxima of the complex and the free reagent is important and suitable for the exact determination of metal ions in their solutions. At pH 1–4, arsenazo (III) reacts with rare earth elements to form colored complexes.

The presence of the arsonic group AsO(OH)2 causes the formation of stable complexes of some metals even in strong acid solutions. The presence of the azo group ensures the color reaction (Marczenko and Freiser 1981).

## Results and discussion

### Characterization

**Fig. 1:**
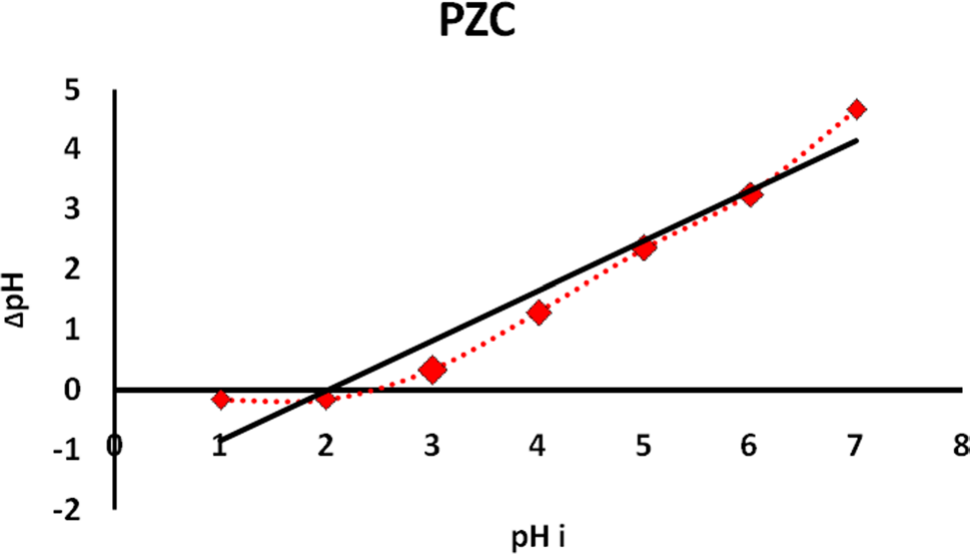
Point of zero charge (PZC) of graphite/alginate adsorbents

Zero charges point (pHpzc), XRD, FTIR, DTA, TGA, and SEM were employed to define the produced adsorbent. The pH value that the total of + Ve and − Ve charges on a solid’s surface equals zero is the zero charges point (pHpzc). While the GRA pH falls below pHpzc, it becomes positively charged, and while the pH rises beyond pHpzc, it becomes negatively charged. Figure 1 shows the difference between the initial and final values versus the initial pH values. The same experimental procedure was shown in De Rossi et al. (2020). The GRA adsorbent’s pHpzc value was 5.0, suggesting that the adsorbent generated was neutral during this pH.

**Fig. 2:**
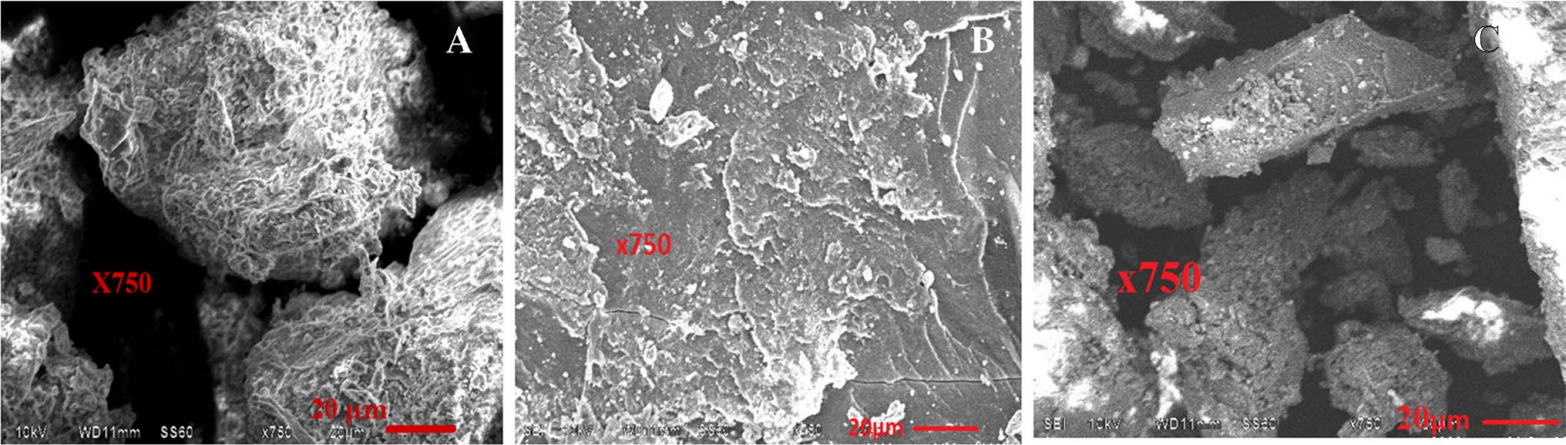
SEM of **A** GRA, **B** GRA loaded with La(III), **B** GRA loaded with Ce (III)

The SEM photographs of graphite/alginate (GRA) adsorbent before and after adsorption were evaluated (magnification was X750, and 20 µm scale) and they are shown in Fig. 2. Before adsorption (Fig. 2A), GRA adsorbents have a well-defined interconnected, unique 3D porous network framework. The 3D porous network can enable metal ion-free diffusion and ensure that metal ions are transported to the adsorbents’ interior structure. After the adsorption of Ce(III) and La(III) (Fig. 2B and C), the GRA surface appeared to be cloudy and dull but at the same time, the structure of the prepared adsorbent remains stable in the adsorption process. The structural stability of the adsorbent is an indispensable part of the application of the adsorbent. Cho et al. (2013) described that the nanosized carbons impregnated in the bead were not visible in the SEM analysis, probably due to the encapsulation of nanosized carbons in the interior space (Cho et al. 2013).

**Fig. 3:**
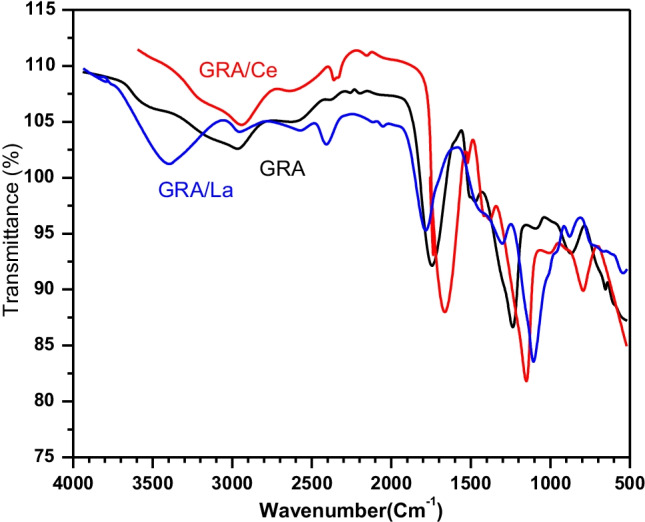
FTIR spectrum for (GRA) adsorbent before and after loading with La(III) and Ce(III)

The prepared adsorbent’s FT-IR spectra were observed before adsorption of Ce(III) and La(III) and after their adsorption to detect the groups responsible for adsorption and the changes that occurred after adsorption (Fig. 3). The existence of a carbonyl C = O stretching vibration group connected with the COO^−^ groups of alginate loaded on the interface of graphite is evidenced by a strong absorption peak at 1655 cm^−1^(Verma et al. 2020). It was noted that the C = O peak was changed through shifting or shrinking due to interaction and chemical bond formation with La(III) (GRA/La beak) and Ce(III) (GRA/Ce beak). The broad absorption band at 3290–2800 cm^−1^ is assigned to O–H stretching vibrations of phenolic or alcoholic functional groups. The absorption at 880–995 cm^−1^ is attributed to R = C–H and R = CH_2_ stretching vibrations that are shifted to a lower frequency because of conjunction with lanthanides metal ions. The sharp medium peak at 2230 cm^−1^ is attributed to a conjugated C≡N (nitrile) stretching vibration.

**Fig. 4:**
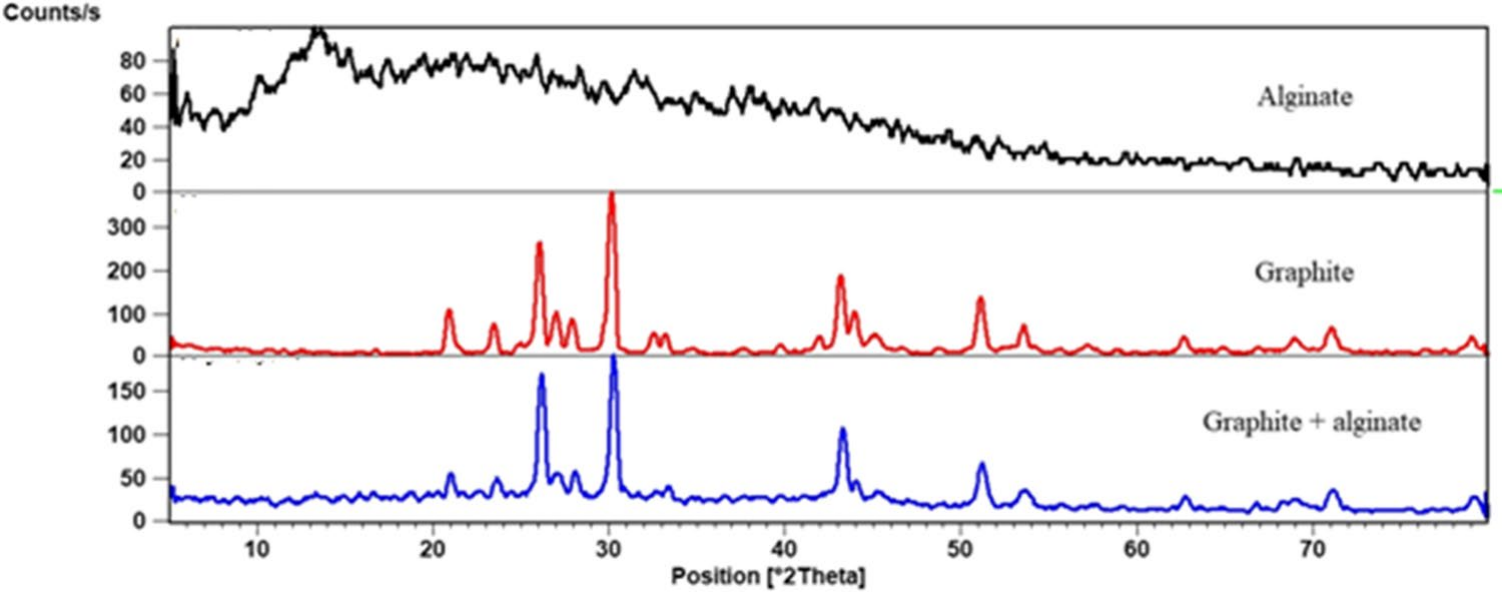
Illustrated the XRD patterns of alginate, graphite, and GRA

XRD patterns of GR, alginate, and GRA are in Fig. 4. The GR pattern showed an intense and sharp peak at 30.3^0^. According to the Bragg equation, this peak corresponded to a d-spacing of 296°A, which confirms the synthesis of GR. In the XRD spectrum of alginate, the broad peak at 13.2° demonstrated the amorphous structure of alginate. Alginate was usually semi-crystalline due to the strong interaction between alginate chains through intermolecular hydrogen bonding (Thakur et al. 2016). The GRA pattern was similar to the GR pattern. This may be due to the low alginate content in the GRA. Because of the low content of alginate in GRA, the XRD spectrum of GRA shows a significant GR diffraction peak; moreover, more information was obtained, which indicated that the GRA had a crystalline structure.

The degree of particle size (*D*) of the prepared material could be calculated from the XRD line extending the curve of the Scherrer formula (Attallah et al. 2019a, b); *D* = Kλ/βcosθ, where D is the crystalline size; λ is the X-ray wavelength (CuKα-1.541836); k is the Scherrer constant (0.89), and β is the angular width of the full width of the diffracted peak at half maximum (FWHM) in the diffraction angle radii (2θ). The average particle sizes were determined from the most intense peaks at 30.12 (2θ). The calculated particle size of GR and GRA was 80 and 90.28 nm respectively.

**Fig. 5:**
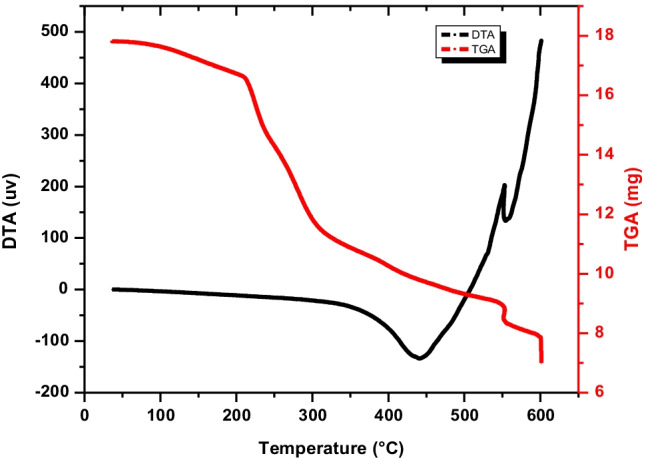
Thermal analysis DTA and TGA of the prepared GRA adsorbent

Thermal analyses of TGA and DTA of GRA adsorbent are illustrated in Fig. 5. According to TGA analysis, the thermal degradation of the synthesized material is usually accompanied by a few main steps. The 1st step includes physicochemical transformation and occurs at low temperatures. The low-temperature mass loss from 30 to 200 °C corresponds to intact water desorption (evaporation of physically absorbed water). The most weight loss was found for the GRA degradation step (200–600 °C). Three degradation peaks (at 220, 480, and 600 °C) are observed. The loss of moisture-bound water in GRA happens around 220 °C and is represented in the curves by a slight weight loss (− 6.7). By raising the temperatures (up to 480 °C) destruction takes place of the less condensed fractions, such as the alginate (organic component) with weight loss (− 40.55%) corresponding to endothermic beak according to DTA analysis at 440 °C. Finally, at about 600 °C, the thermal actions are related to the pyrolysis (crystal dehydration) of the more condensed materials of graphite with weight loss (− 8.6%), and the total weight loss reaches about (55.9%).

The data of surface area is clarified in Table 1. The bulk density evaluates the sample volume, the open as well as closed pores, whereas the apparent density is interested by closed pores and the sample volume. By comparing the bulk density and apparent density for both the materials, it is evident that the open pores in GRA composite will increase. From Table 1, the value of average pore diameter of GRA composite decreases about twice time as the porosity and densities increase for GR sample. This is confirming the increasing the open pores that will occupy and so enhancement adsorption process of the sample. According to the elemental analysis for GRA, the percent of C, O, S, and Na is 94.2, 1.7, 3.01, and 1.087 respectively.

**Table 1:** Surface area and porosity measurements of the prepared samples

Sample	BET m^2^/g	Average pore diameter (nm)	Apparent density (g/mL)	Bulk density (g/mL)	Porosity (%)
GR GRA	8.3 93.7	255.1 110.7	0.8122 3.0430	0.1154 0.5244	30.43 67.37

### Batch technique

#### Effect of pH

**Fig. 6:**
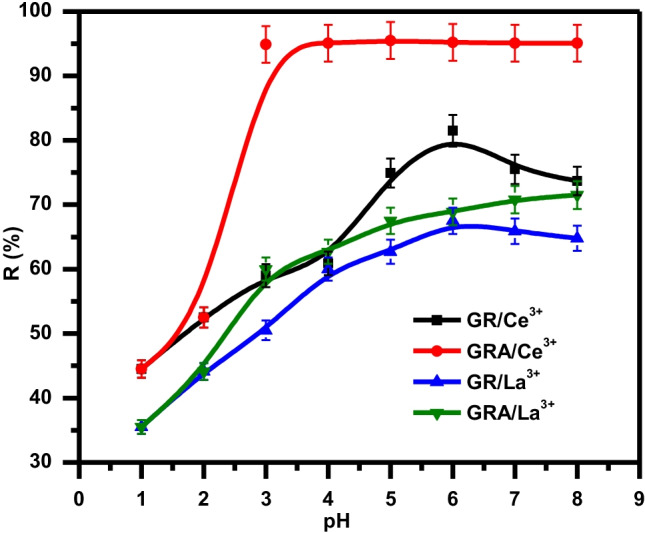
Effect of pH on the removal of La(III) and Ce(III) using GRA and GR

Among the environmental parameters that have much influence on the adsorption process is the pH of the solution. This is for the degree of protonation, metal speciation, and adsorbent surface functional groups that are usually affected by the pH change. Experiments were carried out in the pH range 2–8 to study the effect of the maximum removal efficiency of cerium and lanthanum on the value of pH. Figure 6 illustrates cerium (III) and lanthanum (III) adsorption by GR and GRA as a function of pH. As illustrated, Ce and La adsorption increases rashly with pH from 1 to 5, then stabilizes around pH 6 until pH 8.

The reduction in H^+^ ion interaction with the GR and GRA surfaces can be attributed to the quick increase in uptake quantity with a rise in pH. Because there are more hydrogen ions in the liquid phase vying for sorption sites with cerium and lanthanum ions at low pH, this can result in hydrogen ions preferentially adsorbing onto the adsorbent surface over Ce and La ions, resulting in a reduction in uptake at low pH.

When the pH was raised above pH 5 (low hydrogen ion accumulation), the hydroxyl (OH), carboxyl (COOH), and nitrile (CN) present on the surface, as shown in FTIR analyses, became negatively charged and interfered with the strength of the combination between functional groups of GRA and ions in adsorbate, interfering with the ionic exchange of ions in the bio-adsorbent. These results are confirmed with the result of PZC Fig. 1. Beyond *pH* = 5, the surface becomes negatively charged which increases the opportunity for chelating reaction (via formation of an ionic and covalent bond with metal ions) on GRA.

According to Mahmood et al. (2017), alginate is crucial in the removal of toxic metals in water treatment. Biodegradability, biocompatibility, cost-effectiveness, and environmental friendliness are among its benefits. During the adsorption phase, ion exchange appears to exchange toxic metal ions with sodium or calcium ions. From Fig. 6, it was noted that GRA has a higher R% (98 and 68% for Ce and La respectively) than GR (75 and 63% for Ce and La respectively) so that further batch experiments including kinetic and isotherm were performed using GRA and GR was neglected.

**Fig. 7:**
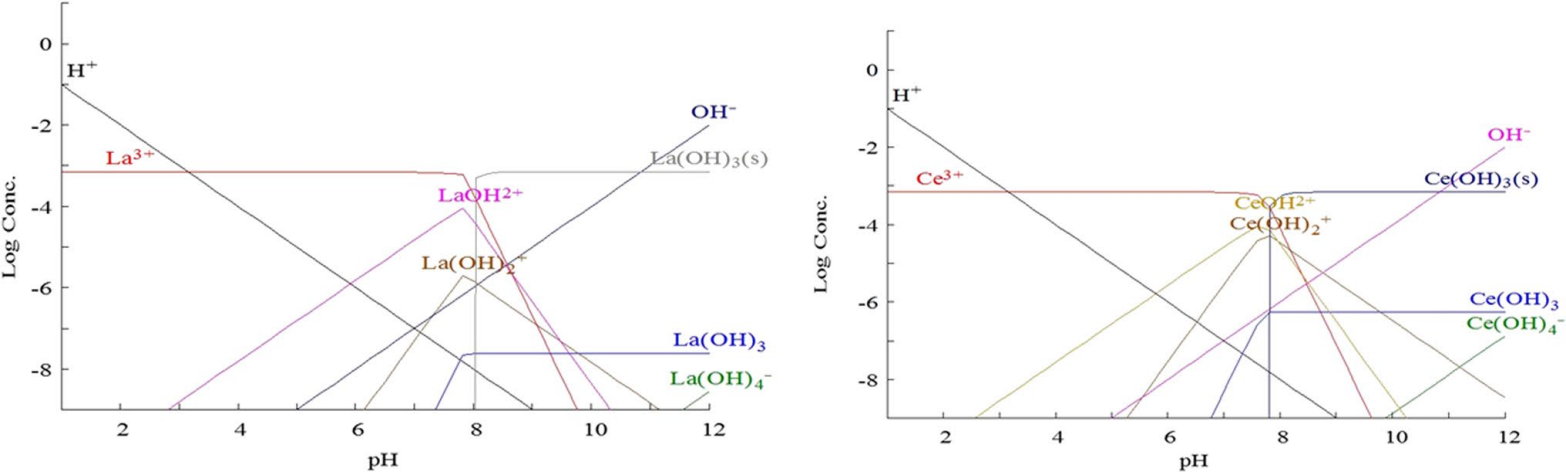
Speciation diagram of La(III) and Ce(III) at different pH range

In particular, from Fig. 7, it has been shown that Ce(III) is present in its stable trivalent form at *pH* ˂ 5 and under this condition, precipitation of Ce(OH)_3_ is negligible (hydroxyl form starts gradually at *pH* = 5). However, as pH increases, precipitation of Ce(OH)_2_ starts at about pH 5.1, and Ce(OH)_2_ starts at about pH 7.9 (Olatunji et al. 2016) Ce(OH) at pH 10.

The lanthanum ion may easily be linked to many water molecules because of its low charge to radius ratio. Bouyer et al. discussed that, during lanthanum ion hydrolysis, hydroxy-ligands replace coordinated water molecules, resulting in the constitution of different hydrolysis species (Bouyer et al. 2006). Figure 7 shows the hydrolysis species as a proportion of pH. The hydrolysis of lanthanum ions is insignificant at acidic conditions (*pH* < 5) as may be shown. Above that critical pH value, hydroxyl form increases gradually, and several hydrolysis products coexist until pH 12 where [La(OH)_4_]^−^ is the only hydrolyzed species in solution. At around pH 6.1, La(OH)_2_ is starting to precipitate and at pH 7.5, La(OH)_3_ is the dominant hydrolytic.

#### Effect of contact time

**Fig. 8:**
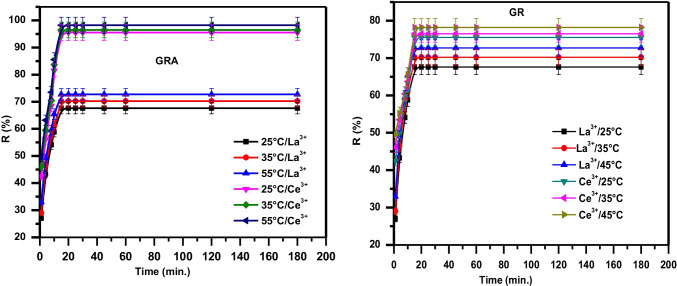
Effect of contact time and temperature on the removal of La(III) and Ce(III) using GRA and GR adsorbents

All experiments were executed in triplicate and the obtained data were scrutinized by variance and stander deviation test using Excel 2010 software and is ranged from 0.37 to 0.51 and 0.19–0.21 for La(III) and Ce(III), correspondingly. The sorption of La(III) and Ce(III) on GRA and GR was investigated by shaking time and temperature at pH 5 and 25, 35, and 55 °C (Fig. 8). At first, the ions selectively adsorbed and occupied the active functional groups on GRA and GR. As the contact time increased, the adsorption sites on the adsorbents were filled. The rate of adsorption slowed slightly before increasing (this is attributed to the availability of large surface areas of the adsorbent) (Mustapha et al. 2019) reached a plateau which indicates that the sorption process is highly time-dependent (Hojati and Landi 2015). The maximum sorption was obtained at pH 5 as a consequence of the experiments and equilibrium was attained after 20 min. All subsequent studies in our work were conducted with a time limit of 20 min.

In the case of GRA (Fig. 8), the removal percent increase with increased temperature. At 25, 35, and 55 °C, the R percent of La(III) reaches around 43.3, 46.5, and 49.2 during the first 5 min, respectively, then increase gradually up to 67.6, 70.2, and 72.7 at 25, 35, and 55 °C respectively at equilibrium time (20 min). While in the case of Ce(III), the R% reach about 56.1, 59.5, and 63.2 at 25, 35, and 55 °C, respectively, at the first (5 min) then increases gradually up to 95.5, 96.5, and 98.2 at 25, 35, and 55 °C, respectively, at equilibrium time (20 min).

While in the case of GR, the R% for La increase gradually up to 62.6, 69.2, and 72.7 at 25, 35, and 55 °C respectively at equilibrium time (20 min). While in the case of Ce(III), the R% reach about 75.5, 76.5, and 83.2 at 25, 35, and 55 °C, respectively, at equilibrium time (20 min). It is clear that GRA shows a super separation between La(III) and Ce(III) more than GR. So GRA was chosen to complete the isotherm experiments.

#### Effect of initial cerium and lanthanum concentration

**Fig. 9:**
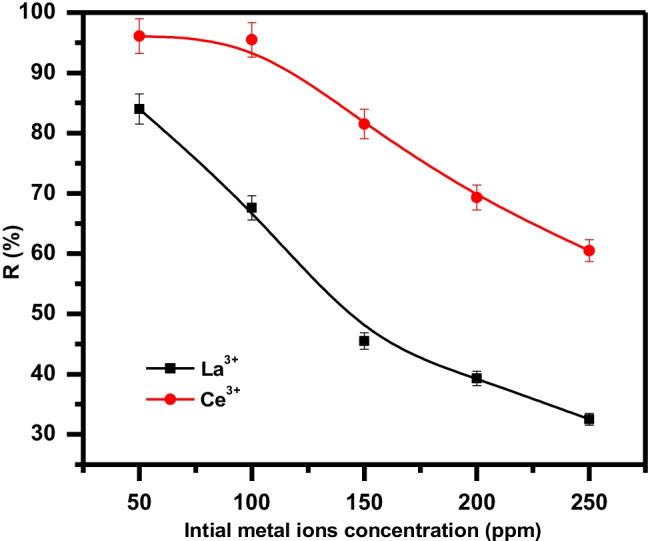
Effect of initial metal ion concentration on the removal of La(III) and Ce(III) using GRA adsorbent

Adsorption assays with starting ion concentrations ranging from 50 to 250 mg/L 1 were carried out to see how the amount of Ce(III) and La(III) uptake was affected by the initial ion concentration. For each experiment, 0.01 g of GRA adsorbent was used at pH 5≈6, 200 rpm shaking speed, 10 ml solution volume, and shaking time of 20 min at ambient temperature of 25 °C. The uptake percent by the adsorbent decreased (from 98 to 60.6% and from 84 to 34% in the state of La and Ce respectively) as the started concentration go up from 50 to 250 mg/L (Fig. 9). This is for the increased competition of the ions for small available sites (Sert et al. 2008). When all the unoccupied sites in the solution–adsorbent interphase have been used, leaving the adsorbate ions in the solution, Ucun et al. (2003) described the drop in efficiency at higher concentrations to adsorbate saturation in the solution–adsorbent interphase. In the current study, similar interpretations can be given to the reduction in uptake amount as initial cerium and lanthanum concentrations varied from 50 to 250 mg/L.

#### Kinetic study

Kinetic modeling for La(III) and Ce(III) adsorption on GRA was investigated using three common models: pseudo-first-order, pseudo-second-order, and intraparticle diffusion kinetic equations. The pseudo-first-order Eq. 3 (Lagergren 1898), pseudo-second-order Eq. 4 (Ho and Mckay 1999), and intraparticle diffusion kinetic model Eq. 5 (Weber and Morris 1963) were shown as follows (Mozaffari et al. 2020a, b, c):

3 $$\mathrm{log}({q}_{e}-{q}_{t})=\mathrm{log}{q}_{e}-\left(\frac{{k}_{1}}{2.303}\right)t$$

4 $$\frac{t}{{q}_{t}}=\frac{1}{{k}_{2}{q}_{e}^{2}}+\frac{t}{{q}_{e}}$$

5 $${\mathrm{q}}_{\mathrm{t}}=\complement +{k}_{p}{t}^{1/2}$$

The amounts of metal adsorbed per gram of GRA (mg/g) at equilibrium and at any moment are q_e_ and q_t_, respectively, and *k*_1_ = rate, constant of pseudo first order sorption (h^−1^), and *k*_2_ (g/mg hour) are the rate determining step for pseudo 2nd order adsorption. The intraparticle diffusion rate constant (mg (g min^1/2^)^−1^) is denoted by k_p_. C is constantly proportional to the boundary layer thickness.

**Fig. 10:**
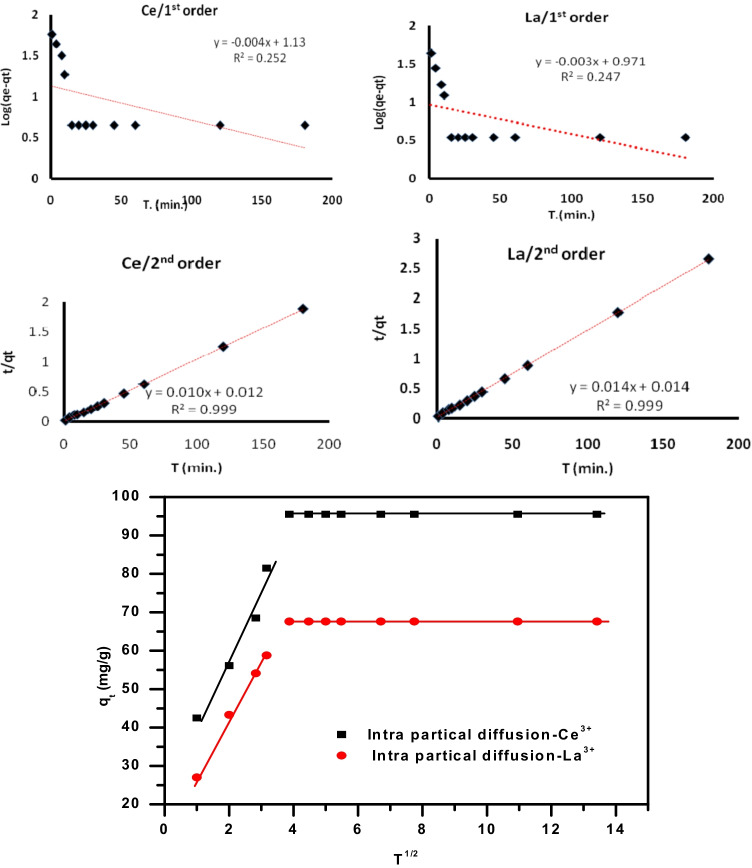
Kinetic study carried out on the removal of La (III), Ce (III) using GRA adsorpent, including pseudo first order second order, and intra-particle diffusion kinetic models

The kinetic characteristics of La(III) and Ce(III) adsorption onto prepared adsorbents are presented in Table 2 and Fig. 10. As seen from the magnitude of correlation coefficients (*R*^2^), for the sorption of La(III) and Ce (III) ions, the pseudo 2nd order exhibited a significant and comparable correlation. The suitability of the proposed kinetic model is confirmed by the comparison of the evaluated adsorption capacity considering the pseudo-second-order equation (q_e_) and that found experimentally (q_exp_). It proposes that the rate-determining step might be a chemical process and the adsorption involves the valance forces throughout sharing of electrons between the La(III), Ce(III) ions, and prepared GRA (Gad and Youssef 2018). The nonlinear relationship between the plotted data of the intra-particle diffusion model and the computed value of “C” (C ≠ 0) implies that this model does not play a significant role in the adsorption of La(III) and Ce(III) ions, and so is not valid for adsorption kinetics.

**Table 2:** Adsorption kinetic parameters of La(III) and Ce(III) adsorption on GRA at 25 °C

Kinetic models	Parameters		
		La(III)	Ce(III)
	q_e_ (exp.) (mgg^−1^)	67.6 ± 1.7	95.5 ± 2.8
Pseudo-first-order equation	q_e_ (Cal.) (mgg^−1^)	2.64 ± 0.07	3.09 ± 0.08
	k_1_ (min^−1^)	0.007 ± 0.0001	0.009 ± 0.0001
	*R*^2^	0.247	0.252
Pseudo-second-order equation	q_e_ (Cal.) (mgg^−1^)	71.4 ± 2.0	100 ± 3.0
	k_2_ (min^−1^)	0.173 ± 0.005	1.429 ± 0.004
	*R*^2^	0.999	0.999
Intra-particle diffusion	k_p_ (mgg^−^1 min^−1/2^)	18.5 ± 2.0	14.09 ± 0.09
	C	21.21 ± 1.04	13.9 ± 1.2
	*R*^2^	0.972	0.966

#### Adsorption isotherms

Isotherm is a crucial criterion because they define how the adsorbate interacts with the adsorbent. Three linear isotherm models were studied to describe the experimental data of La(III) and Ce(III) adsorption on GRA, namely Langmuir types I, II, III, and IV (Eqs. 6, 7, 8, and 9 respectively) (Hamzaoui et al. 2018), Freundlich (Eq. 9) (Moradi et al. 2009), and Dubinin–Radushkevich (D–R) (Robati et al. 2016) (Eq. 10).

6 $$\mathrm{Langmuire\;Type\;I}: \frac{{\complement }_{e}}{{q}_{e}}=\frac{1}{{b \times q}_{\mathrm{max}.}}+\frac{{\complement }_{e}}{{q}_{\mathrm{max}}}$$

7 $$\mathrm{Langmuire\;Type\;II}: \frac{1}{{q}_{e}}= \frac{1}{b*{q}_{m}}\times \frac{1}{{C}_{e}}+ \frac{1}{{q}_{m}}$$

8 $$\mathrm{Langmuire\;Type\;III}: {q}_{e}= {q}_{m}- \frac{1}{b}* \frac{{q}_{e}}{{C}_{e}}$$

9 $$\mathrm{Langmuire\;Type\;IV}: \frac{{q}_{e}}{{C}_{e}}=b*{q}_{m} -b{*q}_{e}$$

10 $$\mathrm{Freundlich\;model}:\mathrm{ log}{\mathrm{q}}_{\mathrm{e}}={\mathrm{logK}}_{\mathrm{f}}+\frac{1}{\mathrm{n}\times {\mathrm{logC}}_{\mathrm{e}}}$$

11 $$\mathrm{D}-\mathrm{R\;model}:\mathrm{ ln}{\mathrm{q}}_{\mathrm{e}}={\mathrm{lnq}}_{\mathrm{m}}-\upbeta {\upvarepsilon }^{2}$$

where Ce (mg/L) and qe (mg/g) denote the leftover concentrations of metals in solution and, correspondingly, the quantity of metal immobilized on the adsorbent at equilibrium. The maximum number of cations per unit mass of bio-adsorbent is q_max_ (mg/g), and b is the equilibrium constant related to the adsorbent-metal ion affinity. K_f_ denotes the adsorbent’s sorption capacity, with n indicating the sorption process’s favorability. n represents the sorption strength (mg L^−1^). (a) If 1/*n* = 1, the adsorption operation is linear and the distribution of metal (M^+^) between the two phases is unaffected by their concentration. (b) If 1/*n* < 1, the process is chemical, and (c) if 1/*n* > 1, the adsorption operation is physical. The 1/*n* number approaches zero as the adsorbent turned to be more diverse (Torab-Mostaedi et al. 2011). If n lies between 1 and 0, this indicates a favorable sorption process (Goldberg 2005). *β* is the Polanyi potential, the average adsorption energy per mole of the adsorbate as it is moved from an infinite to the solid phase.

12 $$\varepsilon =RT\mathrm{ln}\left(1+\frac{1}{{\complement }_{e}}\right)$$

The quantity of mean sorption energy E (kJ/mol) can be determined from the D–R parameter *β* (Eq. 13), where T is the solution temperature (Kel.) and R is the gas constant, which is equal to 8.314 J/mol:

13 $$\varepsilon =\frac{1}{\sqrt{-2\beta }}$$

The mean sorption energy value provides data on chemical and physical sorption. E magnitude can be connected to the reaction mechanism. When E is between 8 and 16 kJ/mol, ion exchange regulates sorption. Physical forces may alter the sorption mechanism when E is less than 8.0 kJ/mol. Chemisorptions, on the other hand, governs the sorption process when *E* > 16 kJ/mol (Hamed et al. 2016; Attallah et al. 2019a, b). The Langmuir adsorption isotherm model was tested using R_L_ to see if the sorption operation was favorable or unfavorable.

14 $${R}_{L}= \frac{1}{1+b{C}_{0}}$$

where *C*_0_ (mg/L) and *b* (L/mg) are initial metal concentration and Langmuir constant, respectively. The *R*_*L*_ cleared the shape of the isotherm to be unfavorable (*R*_*L*_ > 1), linear (*R*_*L*_ = 1), favorable (0 < *R*_*L*_ < 1), or irreversible (*R*_*L*_ = 0). If *R*_*L*_ from 0 to 1, this cleared favorable adsorption.

**Fig. 11:**
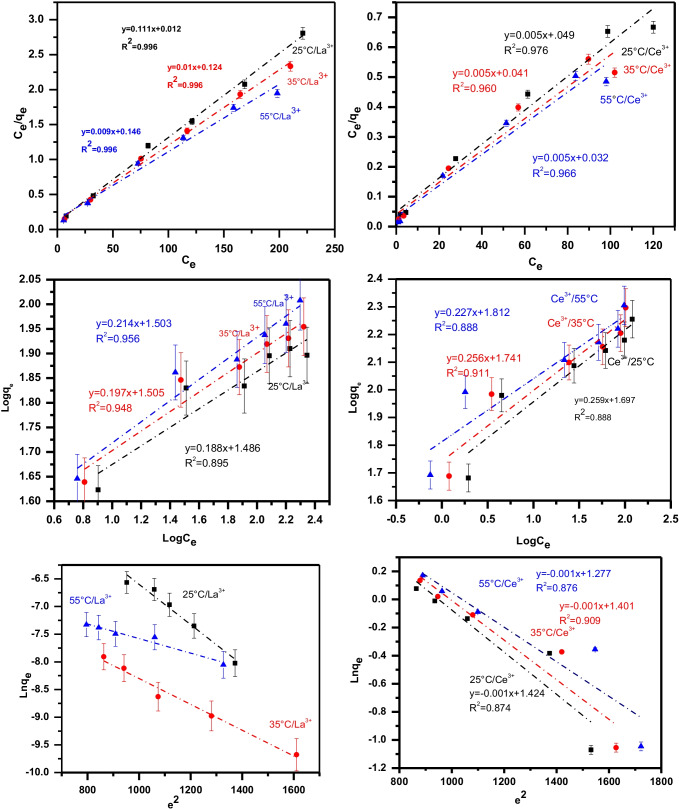
Linear Langmuir (type 1), Freundlich, and D–R isotherm models explanation for La(III) and Ce(III) adsorption on GRA at different temperature

The data is given in Table 3 and Fig. 11. The experimental data for the sorption of La(III) and Ce(III) on GRA can be suited to the Langmuir model (especially the type I model), which assumes that the binding of the adsorbate onto the adsorbent occurs primarily through a chemical reaction and all sites have equal affinity for the metal, based on the calculated correlation coefficients (*R*^2^) for the D–R, Langmuir, and Freundlich isotherms.

**Table 3:** Different isotherm models including Langmuir, Freundlich, and D–R models affection adsorption of La(III) and Ce(III) on the surface of GRA adsorbent at different temperature

Isotherm model	Parameters of different types of Langmuir isotherm models												
	La^3+^							Ce^3+^					
	Temp.(°C)	q_e (exp.)_ mg/g	q_e (calc.)_ mg/g	b (L/mg)	*R*_*L*_		*R*^2^	qe _(exp.)_ mg/g	qe _(calc.)_ mg/g	b (L/mg)	*R*_*L*_		*R*^2^
Langmuir type 1	25	81 ± 2.31	83.3 ± 2.48	0.11 ± 0.003	0.08		0.996	180 ± 5.4	200 ± 6.0	0.102 ± 0.003	0.09		0.976
	35	90 ± 2.56	100 ± 2.88	0.81 ± 0.024	0.01		0.996	198 ± 5.9	200 ± 6.0	0.121 ± 0.004	0.08		0.960
	55	101 ± 2.99	111.1 ± 3.32	0.06 ± 0.001	0.14		0.986	202 ± 6.1	200 ± 6.0	0.156 ± 0.005	0.06		0.966
Langmuir type 2	25	81 ± 2.31	83.3 ± 2.48	0.13 ± 0.004	0.07		0.978	180 ± 5.4	166.6 ± 4.9	0.222 ± 0.006	0.04		0.964
	35	90 ± 2.56	90.90 ± 2.73	0.15 ± 0.005	0.06		0.984	198 ± 5.9	166.6 ± 4.9	0.352 ± 0.011	0.02		0.977
	55	101 ± 2.99	100 ± 2.88	0.15 ± 0.005	0.06		0.967	202 ± 6.1	200 ± 6.0	0.500 ± 0.015	0.01		0.962
Langmuir type 3	25	81 ± 2.31	81.47 ± 2.44	0.13 ± 0.004	0.06		0.937	180 ± 5.4	158.9 ± 4.7	0.254 ± 0.007	0.03		0.828
	35	90 ± 2.56	87.45 ± 2.62	0.15 ± 0.005	0.06		0.947	198 ± 5.9	165.9 ± 4.9	0.355 ± 0.011	0.02		0.820
	55	101 ± 2.99	93.69 ± 2.81	0.11 ± 0.003	0.06		0.878	202 ± 6.1	167.7 ± 5.0	0.622 ± 0.018	0.01		0.789
Langmuir type 4	25	81 ± 2.31	82.32 ± 2.45	0.125 ± 0.004	0.07		0.937	180 ± 5.4	167.1 ± 5.0	0.210 ± 0.006	0.04		0.828
	35	90 ± 2.56	88.38 ± 2.65	0.142 ± 0.003	0.06		0.947	198 ± 5.9	174.2 ± 5.2	0.292 ± 0.008	0.03		0.820
	55	101 ± 2.99	95.76 ± 2.87	0.132 ± 0.004	0.07		0.878	202 ± 6.1	177.3 ± 5.3	0.491 ± 0.014	0.01		0.789
Parameters of Freundlich isotherm models													
		qe _(exp.)_ mg/g	*n*	1/*n*	*K*_*f*_	*R*^2^		qe _(exp.)_ mg/g	*n*	1/*n*	*K*_*f*_	*R*^2^	
Freundlich parameters	25	81 ± 2.31	5.32 ± 0.16	0.18 ± 0.005	4.42 ± 0.13	0.895		180 ± 5.4	3.86 ± 0.11	0.26 ± 0.008	5.46 ± 0.16	0.888	
	35	90 ± 2.56	5.08 ± 0.15	0.19 ± 0.006	4.50 ± 0.14	0.948		198 ± 5.9	3.91 ± 0.12	0.26 ± 0.008	5.70 ± 0.17	0.911	
	55	101 ± 2.99	4.67 ± 0.14	0.21 ± 0.006	4.49 ± 0.13	0.956		202 ± 6.1	4.41 ± 0.13	0.23 ± 0.006	6.12 ± 0.18	0.888	
		Parameters of D–R isotherm models											
D–R parameters		qe _(exp.)_ mg/g	q_m_ (mg/g)	β mol^2^ /kJ^2^	E kJ^/^mol	*R*^2^		qe_(exp.)_ mg/g	q_m_ (mg/g)	β mol^2^ /kJ^2^	E kJ^/^mol	*R*^2^	
	25	81 ± 2.31	51.5 ± 1.5	0.003	12.9 ± 0.4	0.968		180 ± 5.4	785.6 ± 23.6	0.001	22.4 ± 0.7	0.874	
	35	90 ± 2.56	2.55 ± 0.1	0.002	15.8 ± 0.5	0.985		198 ± 5.9	4059 ± 121.0	0.001	22.4 ± 0.7	0.909	
	55	101 ± 2.99	1.89 ± 0.1	0.004	22.4 ± 0.7	0.957		202 ± 6.1	3585 ± 107.4	0.001	22.4 ± 0.7	0.876

According to Langmuir model (type 1), the GRA has great affinity and selectivity for Ce^3+^ (q_m_ = 200 mg/g at all temperature range) more than La^3+^ (*q*_*m*_ = 83.3, 100, 111.1 mg/g at 25, 35, and 55 °C respectively).

As shown in Table 3, the value of R_L_ was found to be 0.08, 0.01, and 0.14 at 25, 35, and 55 °C respectively for La(III) and 0.09, 0.08, and 0.06 at 25, 35, and 55 °C respectively for Ce(III). The calculated R_L_ values indicated that the adsorption of M^+^ on GRA was favorable.

According to the D–R model, the energy value (E) in the case of La(III) adsorption was found to be 12.9 kJ mol^−1^ at 25 °C and increases gradually with increasing temperature, and this gives an indication that at low temperature, the reaction mechanism was carried out via ion exchange reaction of metal ions with divalent calcium and monovalent sodium ions including in the crystal lattice of GRA, while the E reach to about 22.4 kJ/mol at high temperature 55 °C may be due to the low q_m_ value 1.98 mg/g comparing with a huge amount of q_m_ at 51.5 mg/g at 25 °C.

In the state of Ce(III), E was constant values (22.4 kJ/mol) at all temperature range from 25 to 55 °C which give an indication for chemical reaction via complexation or chelating with different functions groups (COO^−^, OH^−^) of alginate on the graphite surface. The energy (E) differential between La(III) (cation exchange reaction) and Ce(III) (chemical reaction) could explain why Ce(III) has a higher R percent than La(III) (III). According to the Freundlich isotherm model, the value of 1/*n* ˂ 1 which approves the adsorption process is chemically controlled.

A comparison of the q_max_ values obtained for the La(III) and Ce(III) adsorption on GRA with those data collected in the literature about the adsorption of La and Ce on different inorganic materials is shown in Table 4.

**Table 4:** A comparison of the sorption capacity obtained for La(III) and Ce(III) on prepared graphite/alginate adsorbent with other adsorbent materials

Adsorbent materials	Sorption capacity (mg g^−1^)		References
	La(III)	Ce(III)	
*Sargassum* polycystum	83.3	------	(Diniz and Volesky 2005)
*Platanus orientalis leaf powder*	28.7	32.65	)Sert et al. 2008(
*Bentonite*	37.03	------	(Chegouche et al. 1997)
*Polypyrrole/sawdust (PPy/SD) composite*	------	6.57	(Olatunji et al. 2018)
*Carbon nanotubes (MWCNTs)*	59	-----	(Alguacil et al. 2020)
*Tangerine (Citrus reticulate) peel*	154.86	152.79	(Torab-Mostaedi 2013)
*Pinus brutia leaf powder*	*22.94*	*17.24*	(Vijayaraghavan et al. 2010)
Iron oxide loaded calcium alginate beads	123.5	-----	(Wu et al. 2010)
Modified Pinus brutia leaf powder	------	62.1	(Ceren et al. 2012)
Bis-picolyamine resin (M4195)	9.85	3.64	(Junior et al. 2021)
Iminodiacetate resin (TP207)	22.6	18.76	(Junior et al. 2021)
Metal organic framework (MOF)	130	-------	(Allahyari et al. 2021)
Zirconium triethylenetetramine (ZrT)	6.07	62.6	(Lankapati et al. 2021)
*Graphite/alginate(GRA)*	86	200	This work

#### Thermodynamic study

Numerous thermodynamic parameters for the present systems were calculated to understand the thermodynamic nature of the sorption process. The temperature-dependent enthalpy (ΔH°), entropy (ΔS°), and free energy (ΔG°) can all be calculated. The Van’t Hoff Eq. (15) allows the analysis of data by plotting ln K_d_ versus 1/T (Rajabi et al. 2019):

15 $${\mathrm{lnK}}_{\mathrm{d}}=\frac{\Delta {\mathrm{S}}^{^\circ }}{\mathrm{R}}-\frac{\Delta {\mathrm{H}}^{^\circ }}{\mathrm{RT}}$$

where K_d_ is the sorption equilibrium constant that can be calculated using Eq. (16).

16 $${K}_{d}=\left(\frac{{C}_{0}-{\complement }_{e}}{{C}_{e}}\right)\left(\frac{V}{m}\right)$$

Calculating enthalpy (ΔH°) and entropy (ΔS°) is made easier with this analysis. Equation (17) cleared how to determine (ΔG°) in the following way:

17 $$\Delta {G}^{^\circ }=\Delta {H}^{^\circ }-T\Delta {S}^{^\circ }$$

The negative (ΔG°) value shows that adsorption occurs spontaneously, and ΔG° decreases as temperature rises. As shown in Table 4, indicated that the adsorption of La(III) and Ce (III) onto GRA is more favorable at a higher temperature. This is for a rise in the numeral of active surface sites available for metal ions, as well as a shortage in the thickness of the boundary layer around the adsorbent, both of which will speed up the mass transfer process (Ma et al. 2010).

The enthalpy change (ΔH°) is a measurement of the energy barrier that the metal ion must exceed during adsorption onto an adsorbent. As shown in Table 5, the positive values of ΔH° (259.4 and 643.2 KJmol^−1^ for Ce(III) and La(III) respectively) give an indication of the endothermic nature of the Eu(III) adsorption process onto GRA (Dolatyari et al. 2016).

**Table 5:** Results of thermodynamic parameters for sorpion of La(III) and Ce(III) on GRA

La(III)	ΔG° KJ mol^−1^	ΔH°	ΔS°	
25 °C	ΔG° KJ mol^−1^	643.2 ± 19.29	2.78 ± 0.08	
35 °C	− 18.2 ± 0.54			
55 °C	− 21.9 ± 0.65			
Ce(III)				
25 °C	− 26.7 ± 0.80	259.4 ± 7.78		112.2 ± 3.36
35 °C	− 75.7 ± 2.27			
55 °C	− 84.9 ± 2.54			

The positive standard entropy change ΔS° (kJ/mol K) may be due to the release of water molecules produced by the ion-exchange reaction between the metal ions and the surface functional groups of GRA, reflects the affinity of the GRA surfaces for the Ce(III) (which has a large entropy value 112.2 kJ/mol K) than La(III) (2.78 kJ/mol K), and this leads to the increase of randomness at the solid/liquid interface during the adsorption process (Moradi and Zare 2011). Also, Ce(III) has a small ionic radius than La(II) so the diffusion of Ce(III) is higher than La(III) and so the degree of disorder of Ce(III) is higher than La(III).

#### Reaction mechanism

In this study, there are two types of the mechanism included, first preparation of GRA adsorbent and the other includes the selectivity of the adsorbent for Ce(III) more than La(III).

**Scheme 1:**
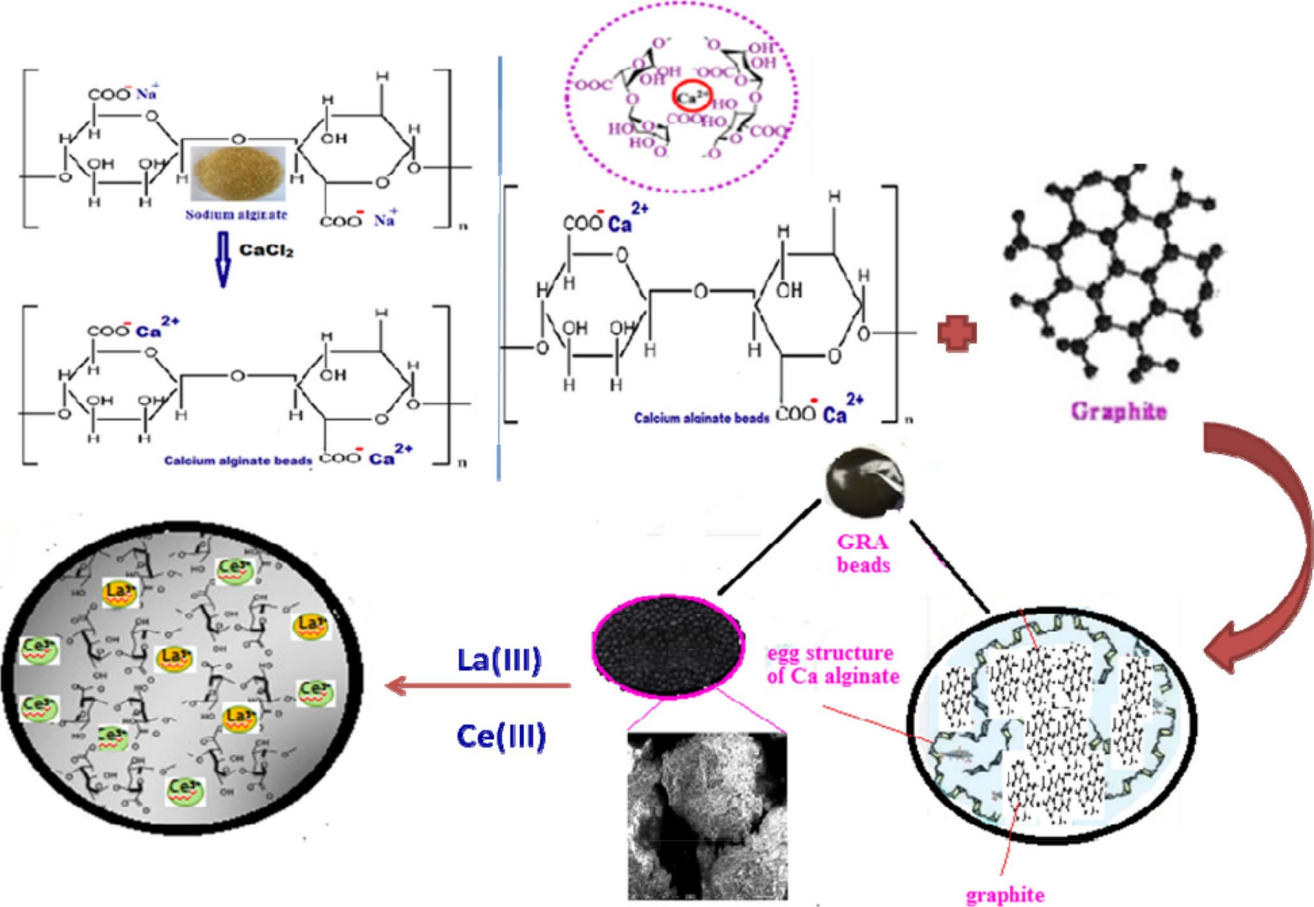
Explain the mechanism of (1) graphite alginate (GRA) preparation, (2) interaction of La(III) and Ce(III) with the prepared GRA

Sodium alginate as a natural anionic polysaccharide mainly possesses abundant free carboxyl groups (-COOH) and hydroxyl (-OH) groups. The carboxyl groups can conjugate with multivalent metal ions, forming egg-box structures (Scheme 1), which have gotten a lot of attention. Firstly, sodium alginate was cross-linked with CaCl_2_ for bead formation and for La(III) and Ce(III) removal from aqueous solution in different conditions. Moradi and Zare reported that the existence of a functional group (COOH) on this adsorbent causes a rise in the negative charge on the carbon surface. The oxygen atoms in the functional groups donate single pair of electrons to metal ions and consequently increase their cation exchange capacity. It can be related also that the carboxylic group can form a complex with the metal ion, leading to an increase in the amount of ion adsorption (Moradi and Zare 2011; Moradi et al. 2010; Moradi 2013).

The graphite material has a 3D layered structure of graphene sheets stacked together by weak (van der Waals) forces after the inclusion of Ca-alginate beads. Graphite has excellent chemical, thermal, and mechanical properties. So, graphite is appropriate material for enhancing the stability of hydrogel composites (alginate) (Verma et al. 2020), the alginate bead was encapsulated with graphite through electrostatic interaction or covalent bond, and GRA adsorbent was formed.

The prepared adsorbent graphite/alginate (GRA) has a great affinity for cerium (98%) more than lanthanum (68%) (although both metal ions have approximately the same characteristics of the lanthanides group) at the optimum condition of temperature (25 °C) and *pH* = 6. This may be related to several reasons:

1. *Electron negativity*: metals with high electron negativity display a strong affinity for the electron clouds of oxygen atoms in COO^−^ and OH^−^ group at the surface of graphite/alginate adsorbent (Fan et al. 2021). According to Pauling, the electron negativity of Ce(III) (1.12) ˃ La(III) (1.10). Also, electron negativity is significant in deciding if a link is ionic or covalent and if it is polarized or non-polarized. The distinction between these two bound atoms in electronegativity. When two M^+^ with a difference in electronegativity ˃ 2 units are bound together, they form an ionic connection. The much more electron-withdrawing element has a negative surface charge, while the weaker electron-withdrawing element has a positive electrical charge. The oxygen of COO^−^ in alginate adsorbent has electronegativity = 3.44. So the bond formation is ionic in nature because the difference between electron negativity between oxygen and Ce (O-Ce) and La (O-La) is 2.32 and 2.30 respectively.
2. *Hydration energy* (*HE*): the lower the (HE) the easier for the metal ion to dehydrate, because dehydration depends on (HE) of the metal ion, Ce^III^ (− 3340 kJ/mol) ˂ La^III^ (− 3285 kJ/mol) (Moldoveanu and Papangelakis 2013). Accordingly, Ce(III) may lose all its hydration water to diffuse through the graphite/alginate surface more easily than La(IIII). So La(III) may undergo a partial ion exchange reaction or chemical complexation reaction but at the same time, GRA displayed more adsorption capacity for Ce(III) (200 mg/g) than La(III) (83.3 mg/g) at optimum conditions. The calculated enthalpy from Table 4 has the same trend of Ce (250.4 kJ/mol) ˂ La(III) (643.2 kJ/mol). The same behavior was observed for Pd, Cd, Cu, and Zn adsorption on the synthetic NaX zeolite (Fan et al. 2021).
3. *Hydrated radius*: the elimination percent increases as the hydrated radius decreases (Fanou et al. 2007). La(III) has reduced mobility in aqueous solution and penetration through GRA adsorbent because of its greater hydration radius (4.52 A°). Ce(III), on the other hand, has higher mobility and permeability due to its lower hydration radius (1.85 A°) (Kennedy and Tezel 2018).

#### Effect of interfering ions

**Fig. 12:**
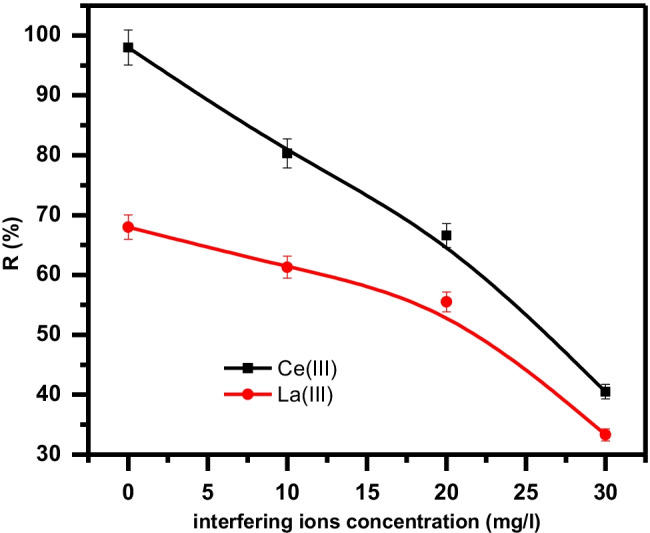
Effect of interfering ions concentration (0, 10, 20, and 30 mg/l) of Na, Ca, and Fe on the adsorption behavior of Ce(III) and La (III)

The effect of competing ions such as Na(I), Ca(II), and Fe(III) on the uptake of La(III) and Ce(III) ions on GRA material was studied as demonstrated in Fig. 12. A mixture of the counterions and metal under investigation La(III) or Ce(III) was prepared. Constant concentration La or Ce (100 ppm) and different concentrations of interfering ions mixture (10, 20, and 30 ppm) were added to 0.01 g GRA at optimum conditions of temp. and pH. It is clear that the percent removal of La(III) and Ce(III) decreased with an increased concentration of competing ions. The significant negative effect of interfering ion (Na, Ca, and Fe) concentrations may be due to either the competition of electrostatic interaction of these ions with the metal under investigation in this study (Kotp 2008) or it may be related to the stereochemical factor. It could possibly be due to Na^1+^ (0.95°A), Ca^2+^ (1.00°A), and Fe^3+^ (0.55°A) that have smaller ionic radius than La (1.15°A) and Ce (1.01°A). It is reflected that the smallest ionic radius implies more molecules could be adsorbed onto the pores of GRA material.

## Conclusion

Graphite was prepared and its surface was chemically modified using alginate to prepare GRA adsorbent. The prepared adsorbent was used for selective separation and removal of La(III) and Ce(III) from an aqueous solution. The adsorption isotherms were well described by the Langmuir and the maximum adsorption capacity reaches 200 and 83.3 mg/g for Ce(III) and La(III), respectively.

The difference between two cations in the affinity towards GRA Adsorbent is strongly related to electron negativity, hydration energy, and hydration radius. The uptake of both Ce(III) and La(III) onto the prepared GRA followed the pseudo second order kinetic model. Ce(III) and La(III) adsorption on the produced GRA followed a pseudo second order model. The sorption operation was spontaneous and endothermic, and they became more active as the temperature rose. This research reveals that GRA can be utilized as an adsorbent to remove lanthanides from a liquid phase.

### Novelty statement

Radioactive waste is radionuclides produced from uranium fission and can be easily discharged into the environment during an accident at a nuclear reactor, such as those that occurred at Chernobyl in 1986, at Three Mile Island in Pennsylvania in 1979, and in 2011 at Fukushima, Japan. In 2009, also discharging for radioactive waste elements such as cesium radionuclide occurred during minor accidents at nuclear power stations in Britain and Germany as well as the USA. So, there have been attempts in developing efficient adsorbents for the removal of the radioactive waste elements from aqueous environments and immobilizing them for safe disposal.

The removal of waste elements (cerium (III) and lanthanum (III)) from the waste stream using synthesized graphite as an inorganic exchanger has never been reported in the literature. Therefore, this study aims to synthesize modified graphite and estimate its potential application in the removal of cerium (III) and lanthanum (III) from the waste stream. The adsorbent was synthesized by thermal annealing technique from easily obtainable and inexpensive raw materials.

The synthesized modified graphite was successfully applied to the removal of cerium (III) and lanthanum (III) from different samples. The modified graphite is shown capacity equal 200 and 83.3 mg/g for Ce(III) and La(III) at 25 °C respectively, from waste water samples, indicating that modified could have wide application prospects in adsorption Ce(III) and La(III) from environmental and waste water samples.

## Data Availability

All authors sure that all data and materials as well as software application or custom code support the published claims and comply with field standards.
