# Correction to: Extraction and separation feasibility of cerium (III) and lanthanum (III) from aqueous solution using modified graphite adsorbent

**DOI:** 10.1007/s11356-022-21676-y

**Published:** 2022-06-27

**Authors:** Maha A. Youssef, Nesreen M. Sami, Hisham S. Hassan

**Affiliations:** Hot Laboratories Center, Egyptian Atomic Energy Authority, Cairo, Egypt

**Correction to: Environmental Science and Pollution Research**

10.1007/s11356-022-20823-9

The correct institution name of the affiliation is revised in the original published proof.

The Original article has been corrected.

